# Unravelling the roles of Autophagy in OSCC: A renewed perspective from mechanisms to potential applications

**DOI:** 10.3389/fphar.2022.994643

**Published:** 2022-10-03

**Authors:** Qiutong Gou, Ling-Li Zheng, Haixia Huang

**Affiliations:** ^1^ Luzhou Key Laboratory of Oral and Maxillofacial Reconstruction and Regeneration, The Affiliated Stomatology Hospital of Southwest Medical University, Southwest Medical University, Luzhou, China; ^2^ Department of Pharmacy, The First Affiliated Hospital of Chengdu Medical College, Chengdu, China

**Keywords:** oral squamous cell carcinoma (OSCC), autophagy, epithelial–mesenchymal transition (EMT), biomarker, diagnosis, treatment, prognosis

## Abstract

Oral squamous cell carcinoma (OSCC) is associated with a low survival rate and a high disability rate, making it a serious health burden, particularly in Southeast Asian countries. Therefore, improvements in the diagnosis, treatment, and prognosis prediction of OSCC are highly warranted. Autophagy has a significant impact on cancer development. Studies on autophagy in various human cancers have made outstanding contributions; however, the relationship between autophagy and OSCC remains to be explored. This review highlights the roles of autophagy in OSCC and discusses the relationship between autophagy and Epithelial–mesenchymal transition. Considering the lack of OSCC biomarkers, we focus on the studies involving OSCC-related bioinformatics analysis and molecular targets. Based on some classical targets, we summarize several key autophagy-related biomarkers with a considerable potential for clinical application, which may become the hotspot of OSCC research. In conclusion, we elaborate on the interrelationship between autophagy and OSCC and highlight the shortcomings of current studies to provide insights into the potential clinical strategies.

## Graphical Abstract

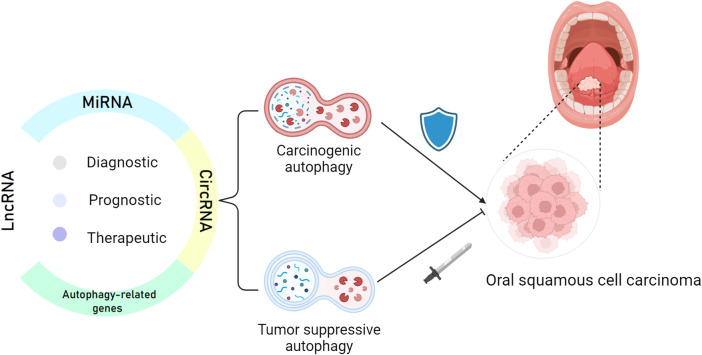

## Introduction

Oral squamous cell carcinoma (OSCC) exhibits a low survival rate and a high deformity rate due to its ability to metastasize and disrupt the upper digestive tract and respiratory tract functions (Ho et al., 2017). The scarcity of clinical diagnosis indices of OSCC often leads to a late diagnosis. Chemoradiotherapy combined with surgery is critical to patients in late stages (stage III and stage IV) or with metastasis (Moratin et al., 2020). However, chemotherapeutic drugs such as cisplatin (CDDP) and 5-fluorouracil fail to produce a satisfactory outcome due to OSCC cell chemoresistance (Li et al., 2019). Moreover, the prognosis of OSCC is mainly predicted by the macroscopic tumor-node-metastasis (TNM)-based staging in clinical practice, where supportive detection at the molecular level is needed. Therefore, new biomarkers are urgently required to improve the diagnosis, treatment, and prognosis prediction of OSCC patients.

Autophagy is an evolutionarily conserved process that leads to the degradation of damaged organelles and recycling of energy sources. Research has contributed to major breakthroughs in autophagy in cancer (Xia et al., 2021). Some critical biological processes in cancer, such as invasion and metastasis, are regulated by the modulation of autophagy and Epithelial–mesenchymal transition (EMT) (Chen H.T et al., 2019). Additionally, the homeostasis of cancer cells is significantly modulated by the crosstalk between autophagy and apoptosis pathways (Das et al., 2021). Hence, targeting autophagy has become a hotspot in OSCC research. However, the roles of autophagy in OSCC remain controversial. On the one hand, the stemness and malignancy of OSCC cells were enhanced by increasing autophagy (Naik et al., 2018). On the other hand, apoptosis was promoted when upregulated autophagy was present in OSCC (Huang et al., 2018). Further discussion on the roles of autophagy in OSCC is required to understand the occurrence and progression of OSCC, which might provide us information for more in-depth studies. The present review summarizes the autophagy-related biomarkers, which possess the potential for clinical applications, and sheds light on the roles of autophagy in OSCC, hence providing clues for new treatment options.

## Targeting Autophagy in OSCC

Autophagy protects cells by maintaining metabolic homeostasis under multiple threats including energy stress and hypoxia stress (Xiang et al., 2020). This biological process is usually presented in three forms, namely macroautophagy, microautophagy, and chaperone-mediated autophagy (Fleming et al., 2022). Among these forms, macroautophagy (commonly known as autophagy), which relies on autophagosomes to deliver autophagic cargo such as damaged organelles and unfolded proteins, is most closely related to cancer progression (Zhang et al., 2018). Classical autophagy involves five consecutive steps: (a) initiation of autophagy, (b) nucleation in vesicles, (c) vesicle formation, (d) docking and fusion, and (e) degradation and recycling of autophagic cargo. Targeting autophagy in some cancers (such as breast cancer) has been proven to be a successful strategy (Xu et al., 2020), providing new inspiration for OSCC studies, which has helped us achieve encouraging results. For instance, cordycepin strengthened the radiosensitivity of OSCC cells by inducing autophagy and apoptosis (Ho et al., 2019). Moreover, enteromorpha compressa extract was predicted to exert a therapeutic effect against OSCC by regulating autophagy and apoptosis (Pradhan et al., 2020). Nevertheless, the specific mechanism through which these compounds modulate autophagy remains to be elucidated. Identification of precise autophagy-related targets and the underlying mechanism/pathway is essential to make targeting autophagy in OSCC a promising approach. Meanwhile, there seem to be divergent views on the role of autophagy in OSCC. Several studies showed that autophagy promotes OSCC progression and inhibiting autophagy may be an effective means to treat OSCC (Chen et al., 2018; Dong et al., 2020), while other studies reached the conclusion that autophagy kills OSCC cells and inhibits cancer metastasis (Yang et al., 2019; Kong et al., 2020). A closer look at the roles of autophagy in OSCC is essential for how to target autophagy (activation or inhibition) in OSCC.

## Autophagy-related biomarkers in OSCC

The identification of specific biomarkers is of great significance for the diagnosis, treatment, and prognosis prediction of cancers. Bioinformatics is a reliable tool to clarify the biological significance of massive biological data, which can help accurately predict biomarkers in cancers. In addition to mining online databases and sequencing alone, genomics, transcriptomics, proteomics, and other omics methods are often combined to improve prediction accuracy (Chen Y et al., 2019). Autophagy-related biomarkers associated with the diagnosis, treatment, and prognosis prediction of OSCC, including autophagy-related genes and non-coding RNAs, such as long non-coding RNAs (lncRNAs), micro RNAs (miRNAs), and circular RNAs (cicrRNAs), have been identified.

### Autophagy-related genes

Eight of the 12 anatomical sites of head and neck squamous cell carcinoma (HNSCC) also belong to OSCC, such as tongue, buccal mucosa, and floor of mouth. Therefore, research on HNSCC has great overlap with that on OSCC, providing meaningful references for OSCC study. Autophagy-related genes are involved in many phases of autophagy, especially in autophagosome formation. Thirty-eight autophagy-related genes, which are differentially expressed in HNSCC tissues relative to normal tissues, were screened out in a recent study mining of HNSCC data from The Cancer Genome Atlas (TCGA). Six genes, namely heat shock protein B 8 (HSPB8), epidermal growth factor receptor (EGFR), integrin alpha-3 (ITGA3), fas-associated protein with death domain (FADD), cyclin-dependent kinase inhibitor 2 A (CDKN2A), and PINK1-Parkin RBR E3 ubiquitin-protein ligase (PRKN), were used to establish a risk model, which proved the practical value of these six genes in predicting HNSCC prognosis. The Kyoto Encyclopedia of Genes and Genomes (KEGG) pathway enrichment analysis indicated that these genes were obviously related to apoptosis, human cytomegalovirus infection, and human papillomavirus infection (Yang et al., 2020). Similar conclusions were obtained in another HNSCC study (Jin and Qin, 2020) using similar methods, wherein seven autophagy-related genes in HNSCC, namely ITGA3, FADD, CDKN2A, HSPB8, NK2 homeobox 3 (NKX2-3), BCL2 antagonist/killer 1 (BAK1), and C-X-C motif chemokine receptor 4 (CXCR4), were screened out. The prognostic value of these genes was confirmed through Kaplan–Meier analysis and receiver operating characteristic curve analysis. Additionally, the top three pathways identified through the KEGG pathway enrichment analysis were the apoptosis-related pathway, human cytomegalovirus infection, and human papillomavirus infection, followed by the epidermal growth factor receptor (ErbB) pathway and hypoxia-inducible factor-1 (HIF-1) pathway. HSPB8, FADD, ITGA3, and CDKN2A are the repeatable targets in HNSCC. In an OSCC study, 37 differentially expressed autophagy-related genes, almost similar to those in HNSCC, were screened out. However, only autophagy-related 12 homolog 12 (ATG12) and BH3 interacting domain death agonist (BID) were confirmed as prognostic biomarkers, unlike those in HNSCC. Interestingly, the KEGG pathway analysis of these genes indicated their enrichment in apoptosis accompanied by the ErbB pathway, platinum drug resistance, and tumor necrosis factor (TNF) pathway (Huang et al., 2021). Moreover, the conclusions of other OSCC studies have been consistent. BAK1, BID, NKX2-3, and sphingosine kinase 1 (SPHK1) were selected as the targets by analyzing OSCC samples, and the downregulation of SPHK1 expression was verified in OSCC pathological tissues through immunohistochemistry (Zhu et al., 2020). These results suggested that HSPB8, FADD, ITGA3, and CDKN2A are repeatable targets in HNSCC, which may also play a role in OSCC, and FADD and NKX2-3 may have high prognostic and therapeutic value in OSCC. However, the bioinformatics analysis of these autophagy-related genes lacks experimental validation (Table 1).

**TABLE.1:** Autophagy-related biomarkers in OSCC.

Biomarker	Type	Role in OSCC	Mechanism/Pathway	Experimental verification	Application	Ref
EGFR	Autophagy-related genes	Carcinogenic	Apoptosis	NO	Prognostic	Yang et al. (2020)
			Human cytomegalovirus infection			
			Human papillomavirus infection			
HSPB8	Autophagy-related genes	Tumor suppressive	Apoptosis	NO	Prognostic	Yang et al. (2020); Jin and Qin (2020)
			Human cytomegalovirus infection			
			Human papillomavirus infection			
PRKN	Autophagy-related genes	Carcinogenic	Apoptosis	NO	Prognostic	Yang et al. (2020)
			Human cytomegalovirus infection			
			Human papillomavirus infection			
CDKN2A	Autophagy-related genes	Tumor suppressive	Apoptosis	NO	Prognostic	Yang et al. (2020); Jin and Qin (2020)
			Human cytomegalovirus infection			
			Human papillomavirus infection			
FADD	Autophagy-related genes	Carcinogenic	Apoptosis	NO	Prognostic	Yang et al. (2020); Jin and Qin (2020)
			Human cytomegalovirus infection			
			Human papillomavirus infection			
ITGA3	Autophagy-related genes	Carcinogenic	Apoptosis	NO	Prognostic	Yang et al. (2020); Jin and Qin (2020)
			Human cytomegalovirus infection			
			Human papillomavirus infection			
BAK1	Autophagy-related genes	Carcinogenic	Apoptosis	NO	Prognostic	Jin and Qin (2020); Zhu et al. (2020)
			Human cytomegalovirus infection			
			Human papillomavirus infection			
NKX2-3	Autophagy-related genes	Tumor suppressive	Apoptosis	YES	Prognostic	Jin and Qin (2020); Zhu et al. (2020)
			Human cytomegalovirus infection			
			Human papillomavirus infection			
CXCR4	Autophagy-related genes	Tumor suppressive	Apoptosis	NO	Prognostic	Jin and Qin, (2020)
			Human cytomegalovirus infection			
			Human papillomavirus infection			
ATG12	Autophagy-related genes	Carcinogenic	Apoptosis	YES	Prognostic; Diagnostic; and Therapeutic	Huang et al. (2021)
			ErbB pathway			
BID	Autophagy-related genes	Carcinogenic	Apoptosis	YES	Prognostic; Diagnostic; and Therapeutic	Huang et al. (2021); Zhu et al. (2020)
			ErbB pathway			
SPHK1	Autophagy-related genes	Tumor suppressive	Apoptosis	YES	Prognostic	Zhu et al. (2020)
			Human cytomegalovirus infection			
			Human papillomavirus infection			
LINC00958	lncRNA	Carcinogenic	Inhibiting miR-4306/AIM2	YES	Therapeutic	Jiang L et al. (2021)
			Inhibiting SIRT1/p53			
AL354733.3	lncRNA	Tumor suppressive	—	YES	Prognostic	Jiang Q et al. (2021)
PTCSC2	lncRNA	Tumor suppressive	—	YES	Prognostic	Jiang L et al. (2021)
UBAC2-AS1	LncRNA	Carcinogenic	—	YES	Prognostic	Jiang L et al. (2021)
LINC01963	LncRNA	Tumor suppressive	—	YES	Prognostic	Jiang L et al. (2021)
MIR600HG	LncRNA	Carcinogenic	—	YES	Prognostic	Jiang L et al. (2021)
AP002884.1	LncRNA	Carcinogenic	—	YES	Prognostic	Jiang L et al. (2021)
RTCA-AS1	LncRNA	Tumor suppressive	—	YES	Prognostic	Jiang L et al. (2021)
AC099850.3	LncRNA	Carcinogenic	—	YES	Prognostic	Jiang L et al. (2021)
AL512274.1	LncRNA	Tumor suppressive	—	YES	Prognostic	Jiang L et al. (2021)
HOTAIR	LncRNA	Carcinogenic	Inhibiting miR-126/PI3K/AKT/GSK-3β	YES	Therapeutic	Sophia et al. (2018)
GACAT1	LncRNA	Carcinogenic	Inhibiting miR-149	YES	Therapeutic	Chen et al. (2021)
LINC01207	LncRNA	Carcinogenic	Inhibiting miR-1301-3p/LDHA	YES	Therapeutic	Lu et al. (2021)
miR-126	MiRNA	Tumor suppressive	Inhibiting PI3K/AKT/GSK-3β	YES	Diagnostic and Therapeutic	Sophia et al. (2018)
miR-149	MiRNA	Tumor suppressive	Inducing tumor-suppressive autophagy	YES	Diagnostic and Therapeutic	Chen et al. (2021)
miR-1301-3p	MiRNA	Tumor suppressive	Inhibiting LDHA	YES	Diagnostic and Therapeutic	Lu et al. (2021)
circ-LRP6	CircRNA	Carcinogenic	Activating-carcinogenic autophagy	YES	Therapeutic	Zhang et al. (2021)
circ-CDR1as	CircRNA	Carcinogenic	Activating-AKT/ERK 1/2/mTOR	YES	Therapeutic	Gao et al. (2019): Cui et al. (2021)
			Inhibiting miR-671-5p			
			Inhibiting miR-876-5p/SLC7A11			

### Autophagy-related non-coding RNAs (lncRNAs, miRNAs, and circRNAs)

**FIGURE 1:**
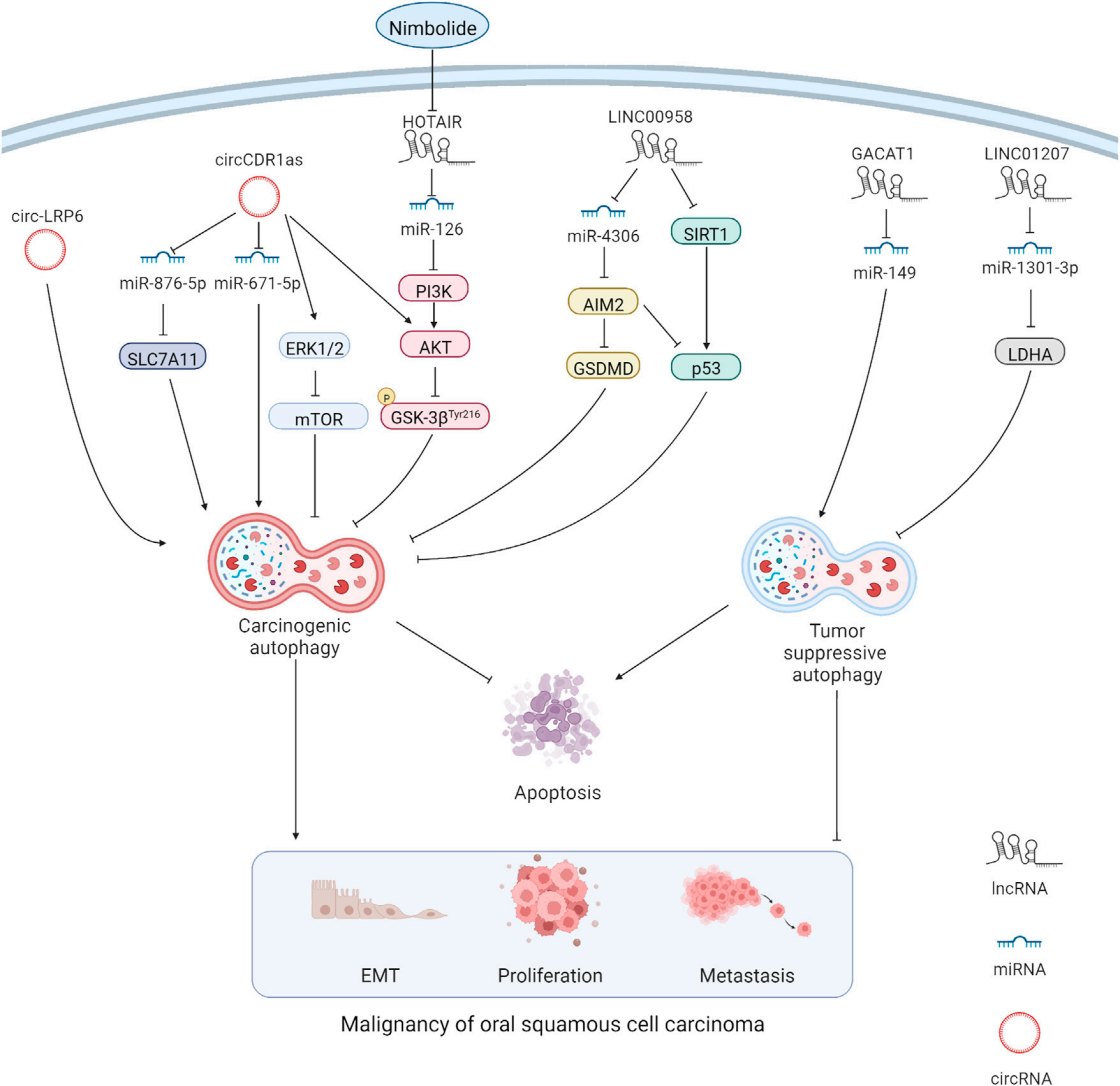
MiRNAs can directly interact with autophagy-related genes. At the same time, lncRNAs and circRNAs act as miRNA sponge through the mechanism of competitive endogenous RNAs, thus affecting the expression of miRNA target genes. Carcinogenic lncRNAs and circRNAs resist apoptosis and promote malignancy in OSCC cells by promoting carcinogenic autophagy and inhibiting tumor suppressive autophagy.

LncRNAs are a type of non-coding RNA with a length of more than 200 nucleotides, and accumulating evidence suggests their indispensable role in cancers (McCabe and Rasmussen, 2021). The lncRNA/miRNA/mRNA axis alters the biological behaviors of cancer cells, such as the ability to undergo EMT and metastasis and show chemoresistance, wherein lncRNAs act as competitive endogenous RNAs (ceRNAs) by sponging miRNAs and changing the expression of their downstream target genes. The similar mechanism exists in OSCC (Figure 1). For example, overexpression of the lncRNA JPX transcript (JPX) was detected in OSCC cells, and JPX silencing could attenuate OSCC malignancy. The internal mechanism might be explained as the binding of JPX to miR-944 and the subsequent augmentation of Cadherin 2 (CDH2) via ceRNAs (Yao et al., 2021). LncRNAs also show carcinogenicity when autophagy is involved. The lncRNA long intergenic non-protein coding RNA 958 (LINC00958) was overexpressed in multiple OSCC cell lines compared with that in normal oral epithelial cells, and increased cell death and inhibition of cell proliferation were observed after LINC00958 silencing, which was accompanied by the decreased autophagy level. Further investigation indicated that LINC00958 sponged miR-4306 to activate the pyroptosis pathway, which was mediated by absent in melanoma 2 (AIM2), and promoted the survival rate of OSCC cells. In addition, sirtuin-1 (SIRT1) expression was downregulated by LINC00958, which, in turn, reduced p53 expression (Jiang L et al., 2021). In a large-scale bioinformatics analysis, a series of autophagy-related lncRNAs, including AL354733.3, papillary thyroid carcinoma susceptibility candidate 2 (PTCSC2), UBAC2 antisense RNA 1 (UBAC2-AS1), long intergenic non-protein coding RNA 1963 (LINC01963), MIR600 host gene (MIR600HG), AP002884.1, RTCA antisense RNA 1 (RTCA-AS1), AC099850.3, and AL512274.1, were predicted to have prognostic values in OSCC, and the results were verified through quantitative reverse transcription polymerase chain reaction (RT-qPCR) and analysis of clinical samples. However, functional annotations were only performed through gene set enrichment analysis (GSEA), and the underlying mechanisms or pathways were not identified (Jiang Q et al., 2021). Although a series of autophagy-related lncRNAs were identified in the aforementioned bioinformatics analysis, the lncRNA HOX transcript antisense RNA (HOTAIR), which can modulate autophagy in various cancers, did not appear in the aforementioned list. HOTAIR overexpression was detected in OSCC cells, and HOTAIR knockdown could suppress the viability, migration, and invasion of OSCC cells by inhibiting autophagy. In addition, the sensitivity of OSCC cells to CDDP was increased, which indicates the therapeutic potential of HOTAIR in OSCC (Wang et al., 2018) (Table 1).

MiRNAs are non-coding, single-stranded RNA molecules with a length of approximately 22 nucleotides encoded by endogenous genes, and they are involved in the regulation of post transcriptional gene expression (Davey et al., 2021). In multiple cancers, the phosphoinositide 3-kinase (PI3K)/protein kinase B (AKT) pathway is obviously regulated by miRNAs, which affects the proliferation and invasion ability of cancer cells (Zhao et al., 2022). The interaction among lncRNAs, miRNAs, and PI3K/AKT pathway-mediated autophagy in OSCC was confirmed in a study using nimbolide, which provided us a new therapeutic target of OSCC, namely miR-126. The upregulation of miR-126 expression was measured after treating SCC-131 and SCC-4 OSCC cell lines with nimbolide. Additionally, protective autophagy was inhibited through inactivation of the PI3K/AKT/glycogen synthase kinase 3 (GSK3) pathway. The apoptosis rate of OSCC cells was also increased (Sophia et al., 2018). Additionally, miR-149 expression was downregulated by the sponge effect of the lncRNA gastric cancer-associated transcript 1 (GACAT1) in OSCC. Furthermore, GACAT1 knockdown promoted autophagy and apoptosis while inhibiting proliferation and metastasis in OSPECAPJ41 and HSC-4CC OSCC cell lines. Interestingly, when the miR-149 inhibitor was applied, the results were reversed, indicating that the lncRNA GACAT1 and miR-149 are promising therapeutic targets in OSCC (Chen et al., 2021). Similarly, in HSC-3 and HSC-4 OSCC cell lines, autophagy and proliferation were inhibited by the effect of miR-1301-3p on the carcinogenic gene lactate dehydrogenase A (LDHA), and apoptosis was promoted. The lncRNA long intergenic non-protein coding RNA 1207 (LINC01207), which is overexpressed in OSCC tissues, could sponge miR-1301-3p to liberate LDHA and cause an unfavorable prognosis (Lu et al., 2021) (Table 1).

CircRNAs are widely existing endogenous non-coding RNAs with a closed ring structure. Unlike traditional linear RNAs, circRNAs are not easily degraded because they are not affected by RNA exonucleases (Kristensen et al., 2022). Similar to lncRNAs, circRNAs act as a miRNA sponge through the mechanism of ceRNA, thus affecting the expression of miRNA target genes (Shi D. et al., 2021) (Figure 1). The circRNA LDL receptor-related protein 6 (LRP6) is highly expressed in OSCC tissues, and its knockdown reduced the autophagy flux and EMT in SCC-15 OSCC cell lines. Notably, the attenuated EMT caused by the knockdown of circ-LRP6 or autophagy related 5 homolog (ATG5) was partially restored by the autophagy agonist rapamycin, revealing the possible interaction between autophagy and EMT in OSCC (Zhang et al., 2021). In addition, the highly-expressed circRNA cerebellar degeneration-related protein 1 antisense (CDR1as) contributed to a higher survival rate of OSCC cells by enhancing autophagy and suppressing apoptosis under hypoxia environment. This phenomenon is associated with miR-671-5p sponging by circCDR1as, which inhibits the mammalian target of the rapamycin (mTOR) pathway and activates the AKT/extracellular signal-regulated kinase 1/2 (ERK1/2) pathway (Gao et al., 2019). Such conclusions provide us with a novel treatment strategy by targeting circCDR1as to reduce the tolerance of OSCC cells to hypoxia. Moreover, circCDR1as modulated solute carrier family 7 member 11(SLC7A11) by sponging miR-876-5p to enhance autophagy-related metastasis and inhibit apoptosis (Cui et al., 2021). This finding confirms the oncogenic role of circCDR1as in OSCC, suggesting that circCDR1as suppression or relieving the inhibitory effect of circCDR1as on downstream targets could be promising in OSCC treatment. At present, there are few studies on circRNA in OSCC autophagy. According to the current results, autophagy-related circRNAs play a carcinogenic role in OSCC; however, more detailed studies are needed to confirm the finding (Table 1). Studies on non-autophagy-related circRNAs have shown that many circRNAs also inhibit OSCC progression; however, the relationship of circRNAs with autophagy remains to be further explored.

## Autophagy as a double-edged sword in OSCC

### Autophagy promotes OSCC progression

The epithelium of the oral cavity mainly originates from the ectoderm, whereas the epithelium of the tongue originates from the endoderm and mesoderm, and these processes are regulated by the Notch pathway. The dysregulation of Notch expression promotes cells to produce stem cell markers such as cluster of differentiation 44 (CD44) and accelerates OSCC progression (Porcheri and Mitsiadis, 2021). Coincidentally, high CD44 levels were detected in OSCC tissues and CDDP-resistant cells under a high autophagic flux, and the inhibition of autophagy could reduce stemness and CD44 expression of OSCC cells (Naik et al., 2018). Furthermore, in OSCC tissues and cell lines, the overexpression of recombinant human retinol-binding protein 1 (RBP1) triggered increased proliferation, migration, and invasiveness of OSCC cells, which was accompanied by abnormally elevated autophagy mediated via the RBP1-cytoskeleton-associated protein 4 (CKAP4) axis; these malignant phenotypes were suppressed after autophagy inhibition (Gao et al., 2020).

Environmental stresses such as hypoxia, energy stress, and damage factors are crucial to cancer progression. The stimulatory effect of autophagy on cancers is partly related to these environmental stresses. For instance, autophagy induced by HIF1-α can help cancer cells resist hypoxia-induced apoptosis. Specifically, the proliferation, migration, and invasion of SCC-9 OSCC cell lines were enhanced by the oncogene special AT-rich sequence-binding protein 2 (SATB2). In addition, hypoxia-induced autophagy and stemness were increased. Intriguingly, SATB2 knockdown reversed this chain action by causing the accumulation of autophagosomes and blockage of the autophagy flux (Dong et al., 2020). Cancer progression requires adequate energy supply. Autophagy can protect OSCC cells by saving energy and recycling sources under starvation. Increased autophagy prevented the OSCC cells from undergoing cell death and apoptosis, whereas 3-methyladenine (3-MA) attenuated autophagy triggered by malnutrition and weakened this protective effect (Abd El-Aziz et al., 2021). In addition, the underlying mechanism of hyperthermia in OSCC was investigated. Hypoxia and starvation caused by hyperthermia could increase the autophagy flux. Interestingly, the inhibition of autophagy in such situations could attenuate cell migration and strengthen hyperthermia-induced apoptosis in OSCC cells (Shi F. et al., 2021). Damage factors such as reactive oxygen species (ROS) can slow down cancer progression. ROS levels increase dramatically under ambient pressure, which may cause serious damage to the cellular structure, a condition known as oxidative stress. Mitophagy is utilized to resist oxidative stress. In OSCC, the terminalia bellirica (TB) extract gallic acid was introduced to promote the accumulation of mitochondrial ROS, causing DNA damage. Consequently, proliferation was inhibited and apoptosis was promoted in OSCC cells. Moreover, autophagy inhibitors 3-MA and chloroquine reinforced TB extract-induced apoptosis in OSCC (Patra et al., 2020). This finding suggests that the inhibition of autophagy might enhance the sensitivity of OSCC cells to anti-cancer drugs.

EMT is a process in which epithelial cells gradually lose their own characteristics and acquire the characteristics of mesenchymal cells. Cells undergoing EMT are usually in a state of transition between epithelium and mesenchymal cells, thereby showing higher apoptosis tolerance and oncogenic potential (Kröger et al., 2019). EMT worsened the lesions by promoting the proliferation and metastasis of cancer cells (Pastushenko and Blanpain, 2019). The expression of the key glycolytic enzyme phosphofructokinase-platelet (PFKP) is significantly increased in OSCC patients, which leads to the increased starvation-induced autophagy and promotes EMT in OSCC cell lines. The abnormally increased autophagy and EMT were blocked after PFKP knockdown (Chen et al., 2018), indicating that a parallel relationship exists between autophagy and EMT in OSCC. More recently, the direct interaction between autophagy and EMT was confirmed by the inhibition of proliferation, migration, invasion, and EMT in OSCC cells following cudraxanthone D (CD) treatment. To investigate the underlying mechanism, the autophagy inhibitor 3-MA and the autophagy agonist resveratrol were applied, which inhibited and promoted EMT of OSCC cells, respectively. Moreover, resveratrol-induced autophagy and the corresponding EMT were suppressed by CD, thus rescuing OSCC cells from acquiring the malignant phenotype, thereby supporting the idea that autophagy promotes EMT in OSCC (Yu et al., 2019). This conclusion is also supported by a more recent study. As previously described, knockdown of circ-LRP6 or ATG5 reduced autophagy and impaired EMT, while application of rapamycin activated autophagy and restored EMT (Zhang et al., 2021).

### Autophagy suppresses OSCC progression

Despite playing a role in promoting OSCC progression, autophagy has been reported to inhibit OSCC progression. The PI3K/AKT/mTOR pathway is closely related to autophagy dysregulation in cancers (Liu et al., 2022). Knockdown of the lncRNA cancer susceptibility candidate 9 (CASC9) in SCC-15 and CAL-27 OSCC cell lines resulted in an obvious downregulation of p-AKT, p-mTOR, p62, and B cell lymphoma/leukemia-2 (Bcl-2), thereby promoting autophagy and apoptosis. Intriguingly, CASC9 knockdown-induced autophagy and apoptosis were reversed by the AKT activator SC79 (Yang et al., 2019). Similarly, a study reported that honokiol could suppress OSCC by increasing autophagy and apoptosis, and inactivation of the AKT/mTOR pathway was also reported (Huang et al., 2018). The relationship between inflammation and cancer has long been studied, and autophagy can inhibit cancer progression through the crosstalk between inflammatory pathways. The nuclear factor-kappa B (NF-kB) pathway in most cancers is overactivated. Toll-like receptor 4 (TLR4) protein promotes cervical lymph node metastasis in OSCC by activating the NF-kB pathway, resulting in a poor prognosis. Autophagy could partially inhibit TLR4-mediated invasiveness by suppressing the NF-kB pathway in SCC-9 OSCC cell lines (Kong et al., 2020). The inverse relationship between autophagy and the NF-kB pathway was further clarified. Ursolic acid was found to increase autophagy and promote apoptosis by inhibiting the AKT/mTOR/NF-kB pathway, resulting in the abrogation of migration/invasion in CA-922 and SCC-2095 OSCC cell lines (Lin et al., 2019). Whether autophagy plays a role in promoting chemoresistance in OSCC is another controversial topic. The sensitivity of OSCC cells (CAL-27 and SCC-9) was enhanced after the inhibition of autophagy, which led the authors to draw the conclusion that autophagy helps OSCC cells acquire chemoresistance (Xu et al., 2017). However, this study used areca nut extract to induce autophagy. Areca nut is the main contributor for OSCC initiation, implying that the increased chemoresistance of OSCC cells may be mainly due to the oncogenic effect of carcinogens, and the role of autophagy in OSCC chemoresistance remains to be explored. More recently, obviously increased autophagy was found in SCC-4 OSCC cells but not in the novel CDDP-resistant OSSC cell line (SCC-4cisR), indicating that autophagy was probably not associated with the acquisition of cisplatin resistance in OSCC cells (Magnano et al., 2021). Probably, the study of inducing drug resistance first, followed by the analysis of the autophagy level in normal and drug-resistant OSCC cells, would yield more convincing results.

## Conclusion and perspectives

This review unraveled the intimate relationship between autophagy and OSCC. On the one hand, autophagy promotes OSCC by maintaining the viability of cancer stem cells, resisting microenvironmental stress, and promoting EMT. On the other hand, autophagy suppresses OSCC by inducing apoptosis and inhibiting invasion and migration of OSCC cells. These contrasting effects may be attributed to differences in the stages of OSCC examined in different studies. Comprehensive studies at specific OSCC stages are needed to explore the exact roles of autophagy in the long process from OSCC initiation to tumor formation and malignant progression. Autophagy-related biomarkers such as autophagy-related genes, lncRNAs, miRNAs, and circRNAs have been reported. These biomarkers regulate multiple biological processes of OSCC through autophagy and exhibit a great potential for clinical application. In terms of diagnosis, the analysis of salivary miRNAs is simpler than that of salivary mRNAs, and miRNAs are not affected by the high digestive sensitivity of RNase, using miRNAs as a biomarker for early diagnosis of OSCC is a feasible option. At the same time, saliva samples are easy to collect and non-invasive for detection, which is easier to be accepted, and is suitable for a wide range of early OSCC screening. Regarding the prognosis prediction, sequencing the tumor tissues resected from patients and establishing and continuously improving the risk model through these autophagy-related biomarkers to predict the 1-, 3-, and 5-year survival rates of patients in combination with clinical indicators, such as tumor grade, may assist clinicians in adjusting treatment plans and communicating with patients. Moreover, further study on the relationship between EMT and autophagy may give instructions to the prediction of OSCC metastasis possibility and lymph node dissection. For OSCC treatment, some existing compounds such as TB and CD have been proven to inhibit OSCC by modulating the autophagy level. Additionally, developing specially designed drugs for the aforementioned autophagy-related biomarkers through molecular docking, high-throughput screening, and molecular dynamic simulation may be promising in improving OSCC treatment. Notably, reliability of some of the biomarkers has not been confirmed through experiments. Experimental verification of the repeatable targets might provide guidance for developing an effective clinical strategy for OSCC treatment.

In summary, this review presents a series of autophagy-related biomarkers and discusses the roles of autophagy in OSCC progression. Further exploration is required in the field of autophagy in OSCC for the improvement of clinical therapy.

## Glossary

3-MA: 3-methyladenine
AIM2: absent in melanoma 2
AKT: protein kinase B
ATG5: autophagy-related 5 homolog
ATG12: autophagy-related 12 homolog
BAK1: BCL2 antagonist/killer 1
Bcl-2: B-cell lymphoma/leukemia-2
BID: BH3 interacting domain death agonist
CASC9: cancer susceptibility candidate 9
CD: cudraxanthone D
CD44: cluster of differentiation 44
CDDP: cisplatin
CDH2: cadherin 2
CDKN2A: cyclin-dependent kinase inhibitor 2 A
CDR1as: cerebellar degeneration-related protein 1 antisense
CircRNA: circular RNA
CKAP4: cytoskeleton-associated protein 4
CXCR4: C-X-C motif chemokine receptor 4
EGFR: epidermal growth factor receptor
EMT: Epithelial–mesenchymal transition
ErbB: epidermal growth factor receptor
ERK1/2, extracellular signal-regulated kinase 1/2
FADD: Fas-associated protein with death domain;
GACAT1: gastric cancer-associated transcript 1
GSEA: Gene set enrichment analysis
HIF-1: hypoxia-inducible factor-1
HNSCC: Head and neck squamous cell carcinoma
HOTAIR: HOX transcript antisense RNA
HSPB8: heat shock protein B 8
ITGA3: integrin alpha-3
JPX: JPX transcript
LDHA: lactate dehydrogenase A
LINC00958: long intergenic non-protein coding RNA 958
LINC01207: long intergenic non-protein coding RNA 1207
LINC01963: long intergenic non-protein coding RNA 1963
LncRNA: long non-coding RNA
LRP6: LDL receptor-related protein 6
MIR600HG: MIR600 host gene
miRNA: MicroRNA
mTOR: mammalian target of rapamycin
NF-kB: nuclear factor kappa B
NKX2-3: NK2 Homeobox 3
OSCC: oral squamous cell carcinoma
PFKP: phosphofructokinase-platelet;
PI3K: phosphoinositide 3-kinase
PRKN: Parkin RBR E3 ubiquitin protein ligase
PTCSC2: papillary thyroid carcinoma susceptibility candidate 2
RBP1: recombinant human retinol-binding protein 1
ROS: reactive oxygen species
RTCA-AS1: RTCA antisense RNA 1
RT-qPCR: quantitative reverse-transcription polymerase chain reaction
SATB2: special AT-rich sequence-binding protein 2
SIRT1: Sirtuin-1
SLC7A11: solute carrier family 7 member 11
SPHK1: sphingosine kinase 1
TB: *Terminalia* bellirica
TCGA: The Cancer Genome Atlas
TLR4: Toll-like receptor 4
TNF: tumor necrosis factor
TNM: tumor-node-metastasis
UBAC2-AS1: UBAC2 antisense RNA 1.
